# OP56 Comparing Large Language Models For Horizon Scanning: A Feature Assessment

**DOI:** 10.1017/S0266462325101141

**Published:** 2025-12-29

**Authors:** Hannah O’Keefe, Chris Marshall

## Abstract

**Introduction:**

Integrating large language models (LLMs) into horizon scanning workflows requires understanding of baseline features, like the ability to extract data and handle noisy data, and contextual understanding to inform considerations for LLM use. We evaluated 25 LLMs to assess their applicability for horizon scanning methods in general and to inform the design and integration strategy of our unit’s advanced horizon scanning system.

**Methods:**

We developed a comprehensive framework detailing 32 features across 10 categories for 25 LLMs. To build this framework, we used ChatGPT-4 to generate a preliminary list of categories, features, and LLMs relevant to HS. We supplemented this with parameters from the 2024 LeewayHertz assessment and finalized it through team consensus. Next, we employed a human-in-the-loop approach utilizing a recursive prover-verifier-chain: Microsoft Copilot>Claude 3.5>ChatGPT-4. Each LLM was assessed for variations in baseline features impacting their applicability in horizon scanning methods and potential integration into our horizon scanning system.

**Results:**

We identified six variable features (19%) across five categories. Nineteen of the LLMs support on-premises or self-hosted deployment. Regarding integration flexibility, only seven LLMs were open source and four lacked strong vendor support. Eighteen models offered a usage-based pricing system, allowing budget tailoring. Five LLMs excelled in handling noisy data, beneficial for horizon scanning methods dealing with diverse information sources. Seventeen models had multimodal capabilities.

**Conclusions:**

Variations in key features among the 25 candidate LLMs affected their suitability for integration into horizon scanning workflows. Units must consider the trade-offs between deployment options, open-source availability, vendor support, pricing models, data handling capabilities, and multimodal features. This extensive framework supports assessment and selection of appropriate LLMs for horizon scanning workflows by filtering models according to these key features.

